# Quantitative in vivo micro-computed tomography for monitoring disease activity and treatment response in a collagen-induced arthritis mouse model

**DOI:** 10.1038/s41598-022-06837-w

**Published:** 2022-02-21

**Authors:** Audrey E. Lord, Liang Zhang, Jamie E. Erickson, Shaughn Bryant, Christine M. Nelson, Stephanie M. Gaudette, Lucy A. Phillips, Annette J. Schwartz Sterman, Soumya Mitra

**Affiliations:** AbbVie Bioresearch Center, Worcester, MA 016015 USA

**Keywords:** X-ray tomography, Rheumatoid arthritis

## Abstract

A painful, chronic condition, Rheumatoid Arthritis, is marked by bone erosion and soft tissue swelling at the joint. As treatments are investigated in pre-clinical models, characterizing disease progression is integral to assessing treatment efficacy. Here, in vivo and ex vivo micro-computed tomography (µCT) are used in parallel with traditional caliper score measurement to quantify physiological changes in the tarsal region in a murine, collagen-induced arthritis model. In vivo imaging methods, which are validated here through comparison to ex vivo and caliper methods, afford longitudinal analysis of both bone and soft tissue through a single image acquisition. This method removes the subjectivity of swelling quantification which is inherently associated with traditional caliper measurements. Histopathology offers an additional assessment of bone erosion and inflammation by providing a microscopic characterization of disease activity. In comparison to untreated animals, daily prednisolone (glucocorticoid) treatment is shown to restore bone volume, as reflected through in vivo and ex vivo µCT images, as well as histopathology. Prednisolone-associated reduction in inflammation is shown through in vivo µCT soft tissue volume measurements, paw caliper measurements, and histopathology. The findings reported here provide a comprehensive validation of in vivo µCT with a sensitivity that enables characterization of pre-clinical disease assessment in response to treatment in a murine, collagen-induced arthritis model.

## Introduction

Rheumatoid arthritis (RA) is a chronic autoimmune disease characterized by synovitis and pannus formation, resulting in cartilage erosion and bone remodeling and destruction. The condition is further associated with pain and progressive loss of joint function. Animal models of RA have been used extensively to interrogate the distinct mechanisms of disease pathology and identify potential biological targets in pursuit of novel therapeutics. Collagen induced arthritis (CIA) is a widely used rodent model of induced arthritis. In this model, the inflammatory response is fueled by the recruitment of neutrophils, macrophages, and T cells. Local fibroblasts, chondrocytes, and synoviocytes cause the destruction of cartilage and bone tissue. These responses to the adjuvant result in two primary identifiable features of CIA: inflammation and bone loss at the joint. The mouse CIA mimics the clinical symptoms, pathological features of synovitis, imaging characteristics, and immunological indicators of human RA^1,2^.

Traditionally, in pre-clinical murine studies, caliper measurements of paw thickness have been used to provide a quick readout of disease activity and are beneficial for their ability to provide data longitudinally pertaining to paw inflammation^3^. Yet, paw thickness does not reveal the underlying structural changes to the bone caused by disease. Assessment of bone morphology by imaging is a more sensitive and translational readout. Radiography is a powerful tool for disease assessment for a vast number of diseases, including RA, providing a visualization of bone architecture changes; however, it is ultimately limited by a 2-dimensional view.

Clinically, computed tomography (CT) has emerged as the preferred method for bone erosion analysis due to its 3-D visualization. CT has also demonstrated increasing sensitivity in quantifying bone erosions in RA patients as compared to magnetic resonance imaging^4^. The advantages offered with clinical CT to document progression of RA are also available with micro-CT (µCT) imaging in preclinical studies^5^. Several studies have demonstrated the value of µCT to quantify bone changes in mouse and rat models of RA^6–8^. However, the dose-dependent effects of this ionizing radiation must be examined prior to using µCT to accurately assess changes associated with disease. Ex vivo µCT has been found to be useful in demonstrating structural bone damage in the Collagen Antibody-Induced Arthritis model (CAIA), as well as prevention of bone degradation via glucocorticoid dexamethasone^9^. While ex vivo µCT can be a powerful tool to assess multiple bone architecture parameters, it lacks the advantage of longitudinal assessment. In contrast, both disease progression evaluation and treatment assessment can be conducted noninvasively using in vivo µCT^10^. In vivo µCT affords the ability to evaluate bone erosion and inflammation through soft tissue volume (STV) changes, while also providing for longitudinal studies, reducing the number of animals needed. This versatile tool can be used to monitor anatomical changes in vivo and findings have demonstrated µCT to be a viable, efficient, and sensitive method to characterize disease progression^11^.

While in vivo µCT bone volume (BV) and STV have been used and validated in the literature as readouts for disease activity^7,12,13^, there has been limited in vivo assessment of disease progression in response to treatment. Previously, in vivo µCT studies have primarily been used to validate µCT against other readouts using naïve animals or to compare naïve and diseased animals. In vivo µCT has been found to be sensitive enough to identify changes in morphology associated with disease, suggesting that any treatment-associated changes could also be identified^11,14^. In this study, we present quantitative assessment of bone and soft tissue changes throughout disease progression and in response to treatment using high-resolution in vivo µCT and correlate them to traditional caliper measurements and histopathology assessment. Further, we examine the level of agreement of data obtained from in vivo µCT and ex vivo µCT to study bone modeling in an animal model.

## Methods

### Materials

Type II Bovine Collagen was obtained from MD Biosciences, Inc. (St. Paul, MN). Complete Freund’s Adjuvant was obtained in a 1.0 mg/mL suspension from BD Life Sciences (Laurence, KS) and concentrated to 3 mg/mL by centrifugation prior to use. Gills 3 hematoxylin was obtained from Richard-Allan Scientific and eosin with phloxine was obtained from Newcomer Supply (Middleton, WI).

### Animals, disease model and treatment

Male DBA/1J mice were obtained from Jackson Labs (Bar Harbor, ME). A total of 22 mice were used, ranging in age from 6 to 8 weeks at the start of the study. A timeline of major events and animal ages is included in Suppl. Fig. 1. All animal study protocols were approved by the AbbVie Bioresearch Center Institutional Animal Care and Use Committee (IACUC). Protocols were conducted in accordance with the Principles of Laboratory Animal Care and monitored by an attending veterinarian. All the animal experiments followed the ethical guidelines of ARRIVE.

Mice were immunized intradermally (i.d.) at the base of the tail with 100 µL of emulsion containing 100 µg of type II bovine collagen dissolved in 0.1 N acetic acid and 333 µg of heat-inactivated Mycobacterium tuberculosis H37Ra. Twenty-one days after immunization with collagen, mice were boosted intraperitoneally (IP) with 1 mg of Zymosan A (Sigma-Aldrich, St. Louis, MO). At the time of the boost immunization, mice were 9–11 weeks old. Following the boost, mice were monitored daily for arthritis. Rear paws were evaluated for pawedema using Dyer spring calipers. Mice were enrolled for the study between days 24 and 28 following the initial collagen immunization at the first clinical signs of disease. At this point in the study, mice were 10–12 weeks old. The day marked by the clinical sign was set as ‘Day 0’ of disease progression. On day 7 of disease progression, the rear paw with the greater caliper score was selected for further analysis and considered the “enrolled” paw. At the termination of the experiment, animals were euthanized with Isoflurane and paws from each group were harvested and stored in 10% neutral buffered formalin for ex vivo µCT and subsequent histopathology.

Following enrollment using clinical indications of disease, the animals were divided into three groups: naïve, untreated, and prednisolone. The ten animals in the naïve group were healthy and untreated. The untreated group was comprised of six diseased animals, which were administered phosphate-buffered saline. The prednisolone group was comprised of six diseased animals treated with late therapeutic dosing beginning on day 7 following enrollment, including 3 mg/kg oral daily dosing of prednisolone (generated by AbbVie Inc.).

An additional group of five age-matched male DBA/1J naïve mice were used to analyze the effects of ionizing radiation. The Day 0 in vivo µCT images of these naïve mice combined with the aforementioned naïve group were used in comparison with diseased mice for analysis. Blood samples were collected from the tail vein for hematological analyses at 0, 7, 14, and 28 days. The white blood cells, platelets, red blood cells, lymphocytes, and neutrophils were measured with the Sysmex Hematology Analyzer XT-2000i (Lincolnshire, IL).

The analysis of this study included data from caliper measurements, in vivo µCT soft tissue volume (STV) and bone volume (BV) measurements, ex vivo µCT BV measurements, and histopathological findings, all from the enrolled mouse rear paw. Each of these measurements pertain to the tarsal region.

### Caliper scoring

Caliper scoring is a commonly used standard metric for disease progression. To measure inflammation in the paw, daily hind paw caliper measurements were in the metatarsal taken for each mouse. Due to heterogeneities in disease induction, the most inflamed paw from each animal based on caliper scores was enrolled and imaged weekly for 21 days.

### In vivo µCT

In vivo µCT images of the hind paws were acquired on days 0, 7, 14, and 21. Using the MILabs U-CT platform (Utrecht, Netherlands) with a nominal resolution of 4 µm, images were acquired with the following settings: X-ray voltage 50 kV, 400 µm aluminum filter, current 0.21 mA, 1 projection per step, step size 0.375°, and 75 ms integration time. The total X-ray dose during one scan was 1100 mGy. The images were then reconstructed using Hann filtered back-projection algorithm (MILabs Reconstruction software; MILabs).

**Figure 1:**
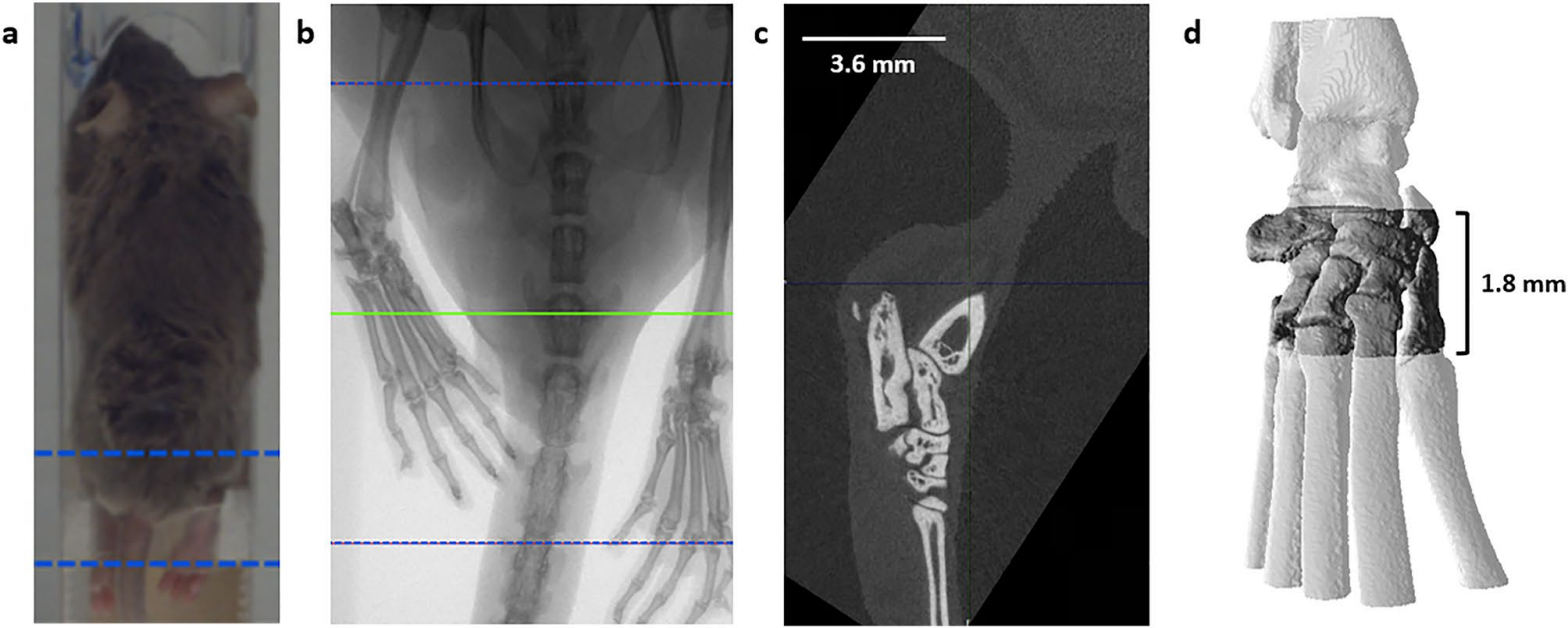
(**a**) In vivo imaging affords longitudinal assessment of the hind paws in the murine CIA model. (**b**) The acquired image is reconstructed using MI Labs software (https://www.milabs.com/u-ct/; Version: 12.0). (**c**) Following reconstruction, the image is reoriented using VivoQuant software (http://www.vivoquant.com/, Version: VivoQuant™ 2020) such that the metatarsals are vertically aligned and orthogonal to the cross-sectional plane in the z-direction. (**d**) The ROI, traversing 1.8 mm vertically, is selected in VivoQuant software by noting anatomical tarsal features.

The mice were anesthetized using isoflurane and laid in the imaging bed in a prone position (Fig. 1a). Images were taken of the hind paws and reconstructed with an 18 µm voxel size and analyzed using VivoQuant (Invicro, Boston, MA) (Fig. 1b). Images of enrolled paws were analyzed and both hind paws of the naïve group were analyzed. For µCT analysis, the image was first reoriented such that the metatarsals were vertically aligned along the z axis and orthogonal to the x–y plane (Fig. 1c). The volume of interest (VOI) was then defined as the region starting at the navicular (central tarsal) bone, excluding the talus, and proceeding distally for 100 slices (1.8 mm) as depicted in Fig. 1d. This region includes the navicular bone and 1st through 4th tarsals (medial, intermedial, and lateral cuneiform; and cuboid bones)^15^. The image was cropped such that the proximal slice in the x–y plane was the slice before reaching the central tarsal bone and the VOI traversed 100 slices distal to that slice. With 18 µm voxel size, 100 slices accounted for a 1.8 mm length for the VOI. The same dimensional VOI was used for ex vivo µCT imaging, the data from which was used for validation of the accuracy of in vivo µCT. Frequency histograms of the density of this region were used to determine appropriate threshold to segment bone and soft tissue. A representative histogram is shown in Suppl. Fig. 2. The 3-D VOI tool available in VivoQuant was used to threshold the image, with all voxels greater than 3500 Arbitrary Units (AU) accounting for bone. In this same VOI, soft tissue was also segmented through a similar manner. Because the intensity of soft tissue is closer to that of air, a Gaussian filter with a standard deviation of 0.1 mm was applied to improve ease of segmentation. Next, the soft tissue was selected with a range of -300 to 1500 AU using the thresholding tool. Pmod software (PMOD Technologies, Zurich, Switzerland) was used to generate 3-D rendered image of the bone from in vivo µCT images.

### Ex vivo µCT

Ex vivo µCT images of the tarsal region were taken following collection of the paws on Day 21. Hind paws were removed at the middle of the tibia/fibula and stored in 10% neutral buffered formalin. Paws were imaged using a Scanco µCT (Scanco Medical AG, Micro-CT40) at 55 kVp and 145 µA, utilizing the high-resolution setting (1 projection per step, step size 0.18°, 300 ms integration time). A Shepp and Logan filter was used for acquisition. These settings resulted in a nominal resolution of 8 µm. Image reconstruction results in isotropic voxels with a width of 18 µm. A cylindrical VOI was manually drawn from the proximal junction of the calcaneus and navicular bone and extending into the tarsals for a fixed height of 100 slices in the mouse (1.8 mm). The VOI used for ex vivo analysis is the same VOI as is used for in vivo analysis and can also be visualized in the 3-D rendering shown in Fig. 1d. 3-D quantification was performed by Scanco AG analytical software for bone volume. Bone volume (BV) measurements from these images were compared to the Day 21 in vivo µCT measurements to assess the correlation between the two methods.

### Histopathology

On day 21, the animals were sacrificed, the hind paws were harvested just proximal to the tarsal joints, immersion fixed in neutral buffered formalin, decalcified in Cal-Rite, and processed in paraffin for histopathological evaluation study. Formalin-fixed tarsal joints were sectioned and stained with Gills 3 hematoxylin and eosin with phloxine. 5 µm thick tissue sections were mounted on slides and scanned using a digital whole slide scanner (Pannoramic 250 Flash III, 3D Histech, Hungary).

Histopathology evaluation included qualitative and semi-quantitative assessment for the presence of inflammation, pannus formation, and bone destruction using a 0–4 scale: 0 = none present, 1 = mild (small foci of inflammation, pannus and bone destruction), 2 = moderate (more numerous, larger foci of inflammation, pannus, and bone destruction), 3 = marked (regionally extensive areas of inflammation, pannus, and bone destruction), 4 = severe (severe diffuse inflammation, pannus, and bone destruction).

### Statistical analysis

Summary statistics are reported as the mean and the standard deviation for each group. Paw swelling summary statistics are also reported as mean and standard deviation. Significant differences between groups were defined as P < 0.05. A two-way ANOVA was used with caliper measurement data, mixed-effect analysis was used with in vivo µCT data, and a one-way ANOVA was used with ex vivo µCT data. For histopathology, differences between groups were assessed using a non-parametric Kruskal–Wallis test followed by Dunn’s multiple comparison tests, comparing each treatment group to the untreated control group. Statistics were generated using Prism software (GraphPad, San Diego, CA).

## Results

**Figure 2:**
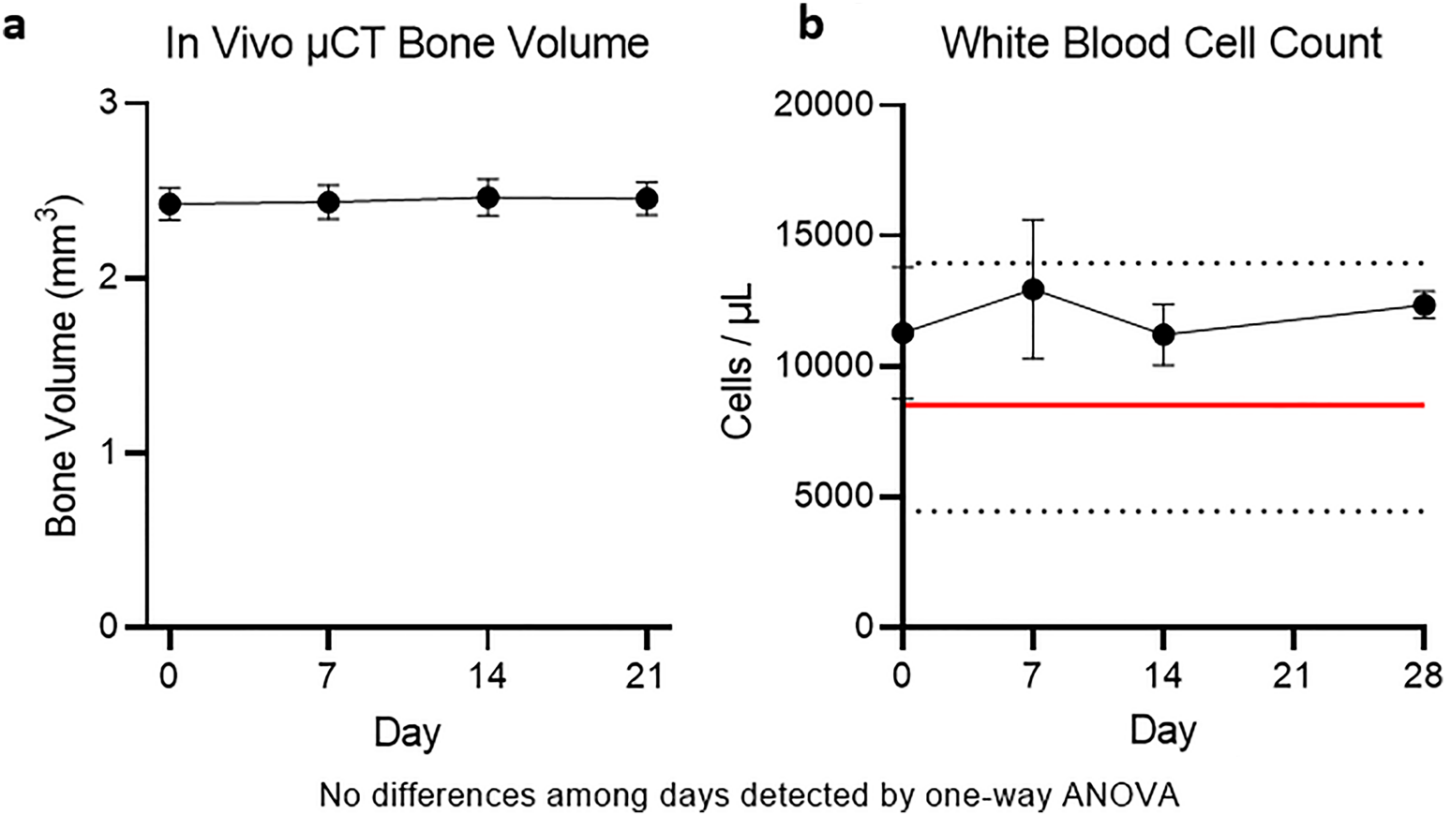
In vivo µCT imaging of five naïve mice shows no imaging-induced effects on (**a**) bone volume, (**b**) or white blood cell count. Horizontal black dotted lines represent a standard reference range of values for C57BL/6 mice from Charles River Laboratories (Wilmington, MA) and horizontal red lines indicate the mean values from C57BL/6 and DBA/1J mice as provided by Jackson Laboratory (Bar Harbor, ME). Standard mean values for DBA/1J mice were provided by the vendor, Jackson Laboratories. No statistical differences among time points were detected by a one-way ANOVA.

Reports in the literature associating ionizing radiation with dose-dependent effects on both blood components and bone necessitate verification that the X-ray dose used in vivo does not impose such effects^16,17^. Here, effects of ionizing radiation are investigated using naïve animals. Naïve animals were imaged using in vivo µCT at Days 0, 7, 14, and 28, and showed no statistically significant change in bone volume or white blood cell counts (Fig. 2 and Suppl. Fig. 3) (see ESM). Histopathology assessment also did not reveal any differences in bone architecture in the irradiated samples (data not shown). Consistency in these values demonstrated toleration of the X-ray dose given as a result of the in vivo µCT scanner settings.

**Figure 3:**
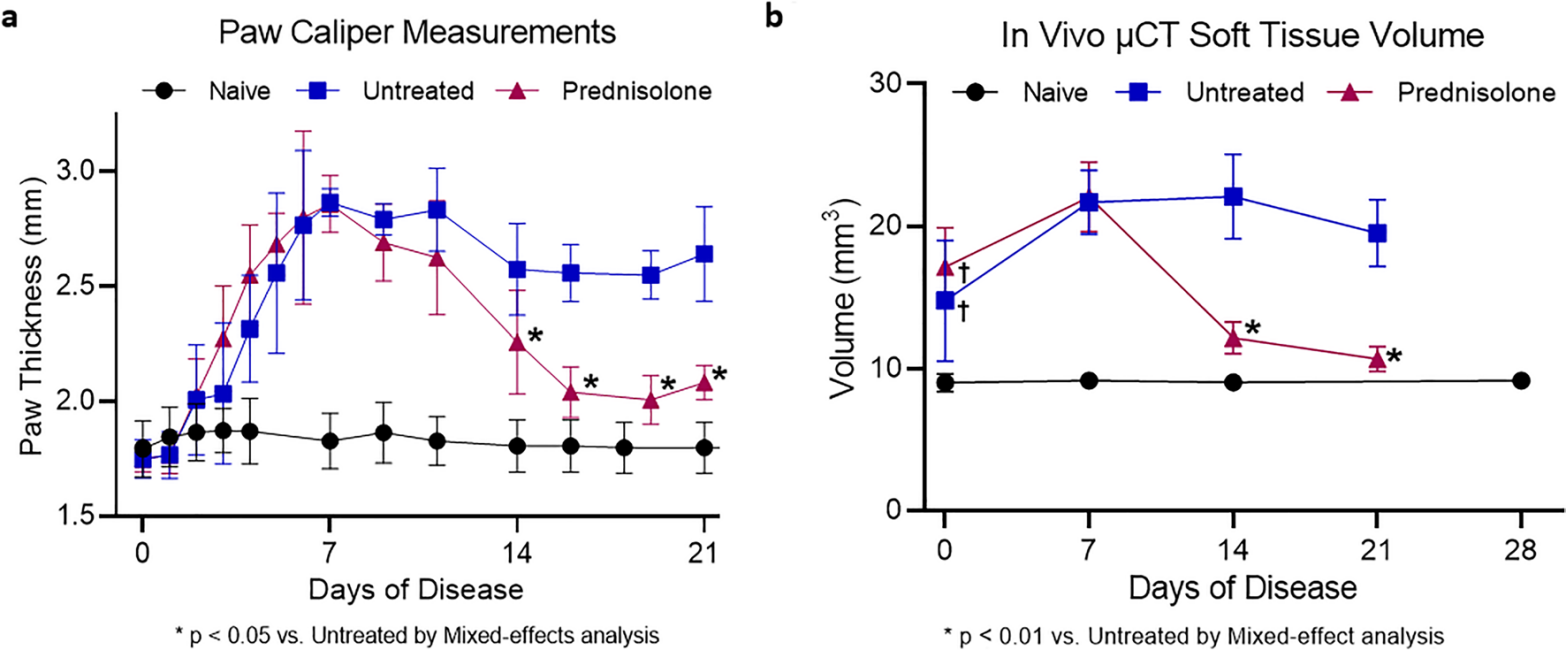
(**a**) Paw caliper measurements provide a quick readout of paw inflammation longitudinally, shown here in terms of change in paw thickness with n = 15 for the naïve group and n = 6 for the untreated and prednisolone groups**.** (**b**) In vivo µCT imaging measures the volume of soft tissue in the tarsal region as a marker of paw inflammation. This readout is expressed here in terms of soft tissue volume longitudinally, where the untreated and prednisolone groups have n = 6. The naïve group has n = 15 on Day 0 and n = 10 on Days 7, 14, and 21.

The paw thickness as measured by caliper scores, as shown in Fig. 3a, demonstrated an increase from Day 0 through Day 7 in both diseased groups. Based on the caliper measurements, the animals reach peak inflammation on day 7 at which point the treatment with Prednisolone is initiated. Day 7 Prednisolone had no effect on the treatment group from Day 0 to Day 7 as treatment was dosed beginning on Day 7. Caliper measurements of the prednisolone-treated group demonstrated a statistically significant decrease in paw thickness on Days 14 and 21 compared to the untreated (Day 21 treated 2.08 ± 0.08 mm vs untreated 2.6 ± 0.2 mm, p = 0.0008) suggesting a therapeutic response resulting in reduced inflammation. However, caliper scores, though correlated to disease activity, are not directly informative of any disease or treatment-induced effect on bone.

Inflammation was monitored through STV as measured through thresholding within the tarsal region VOI. At Day 0, the inflammatory component of the disease is already apparent, as evidenced by a greater STV in the two diseased groups as compared to naïve (p < 0.0001). In vivo STV data agree with paw caliper measurements, where a marked increase in volume in CIA mice relative to naïve control was observed through the first week of disease, followed by a reduction in STV with treatment (Fig. 3b). Prednisolone treatment resulted in decreased STV as compared to the untreated group (Day 21 treated 10.7 ± 0.8 mm^3^ vs untreated 19.5 ± 2.4 mm^3^, p = 0.0004).

**Figure 4:**
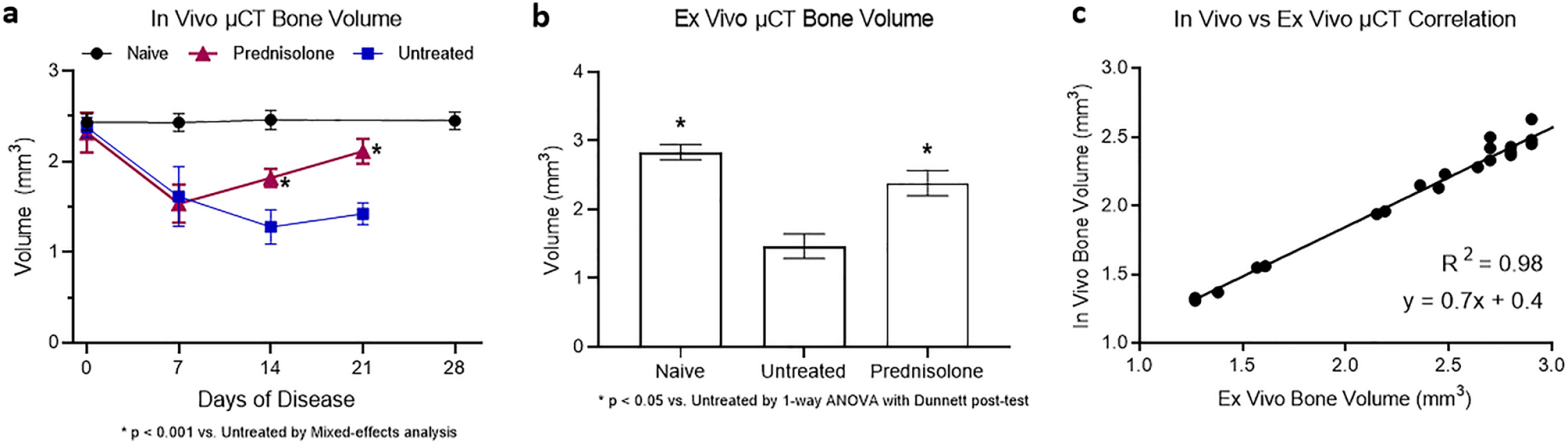
(**a**) In vivo µCT imaging measures tarsal region bone volume over time with n = 20 for naïve and n = 6 for other groups, (**b**) Ex vivo µCT imaging measures tarsal region bone volume with n = 10 for naïve and n = 6 for other groups. The same animals used for in vivo imaging were subsequently used for ex vivo imaging on day 21 of disease. (**c**) In vivo and ex vivo imaging-derived bone volume measurements of the tarsal region are correlated here, including animals from all groups, totaling n = 21. The linear regression performed indicates the correlation has an R-squared value of 0.98.

**Figure 5:**
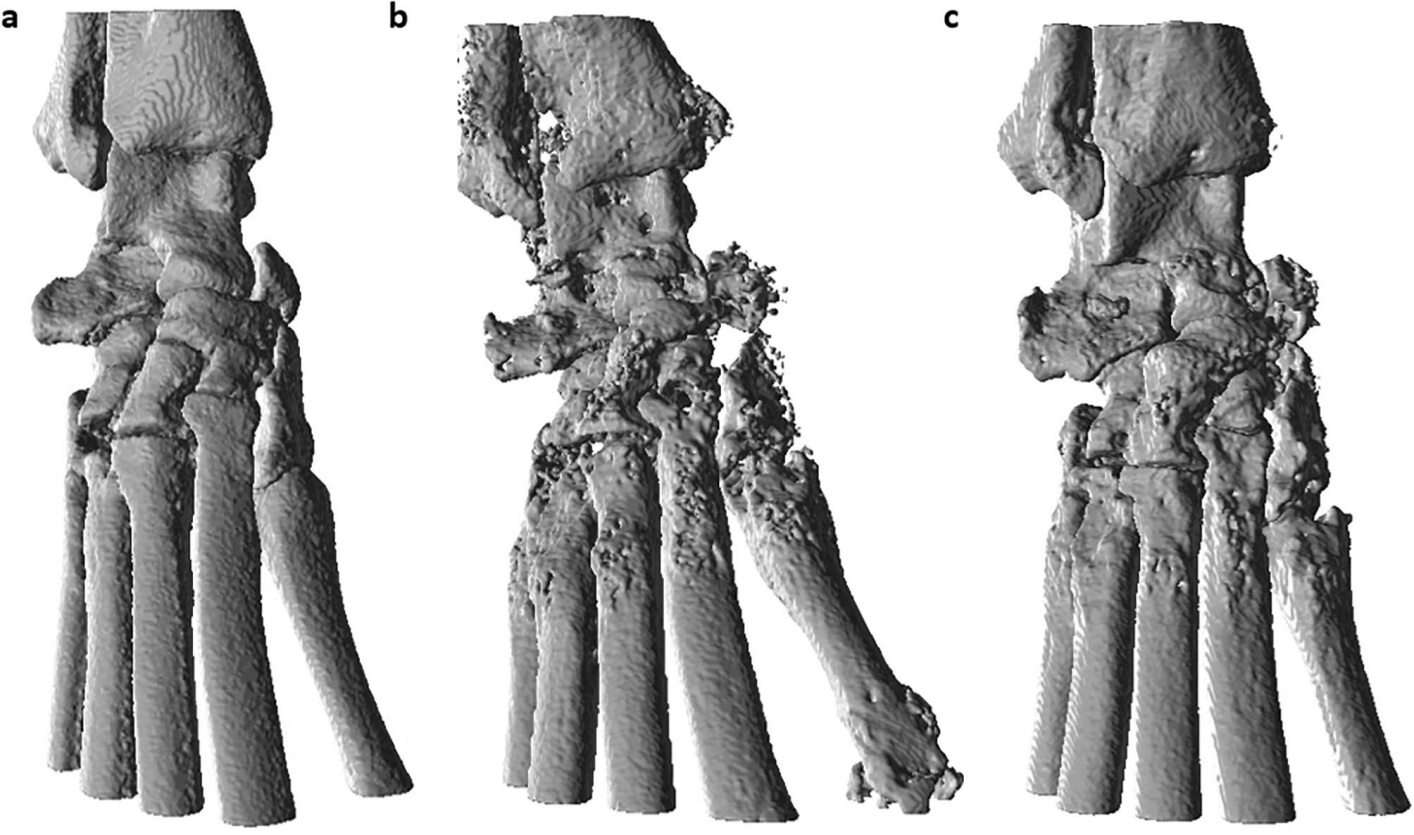
Representative in vivo µCT images from each group have been reconstructed in 3-D here, where (**a**) shows a naïve paw, (**b**) shows an untreated paw, and (**c**) shows a treated paw. A threshold of 3500 AU was used to generate these images in Pmod software (https://www.pmod.com/, Version 4.3).

Tarsal bone volume data were collected to assess disease-associated bone erosion. The pattern as seen in caliper measurements of worsening disease through the first week, followed by improvement in the treatment group, was mimicked by the in vivo and ex vivo BV data. As shown in Fig. 4a, disease progression resulted in decreased BV in both diseased groups, followed by an increase in BV in the treatment group as compared to the untreated group (Day 21 treated 2.1 ± 0.1 mm^3^ vs untreated 1.4 ± 0.1 mm^3^, p < 0.0001). Further, the bone volume of the prednisolone treatment group increased from Day 7 to Day 21 (p = 0.002), demonstrating an effect greater than simply protection against worsening disease. Ex vivo BV data allows for confirmation of the in vivo imaging analysis methods. Figure 4b demonstrates the significant increase in BV on day 21 in the prednisolone treatment vs. untreated group (p < 0.0001). Further, the Day 21 ex vivo BV data agree well with Day 21 in vivo BV data (R-squared value = 0.98) (Fig. 4c). While the ex vivo and in vivo data sets have a highly linear correlation, the equation of the regression line is y = 0.7x + 0.4, where y is the in vivo data and x is the ex vivo data. This 30% difference in slope from 1 may be due to the spacing between the tarsal bones changing postmortem and during the processing steps prior to being mounted onto the Scanco µCT scanner for ex vivo imaging. 3-D reconstruction of representative in vivo µCT images from each group on Day 21 are shown in Fig. 5. In comparison to a naïve sample with a smooth surface (Fig. 5a), these images qualitatively demonstrate disease-driven bone erosion (Fig. 5b), allowing visualization of the rough, pitted surface. Figure 5c shows improvement in response to prednisolone treatment without a return to a “normal” naïve state (Fig. 5c).

**Figure 6:**
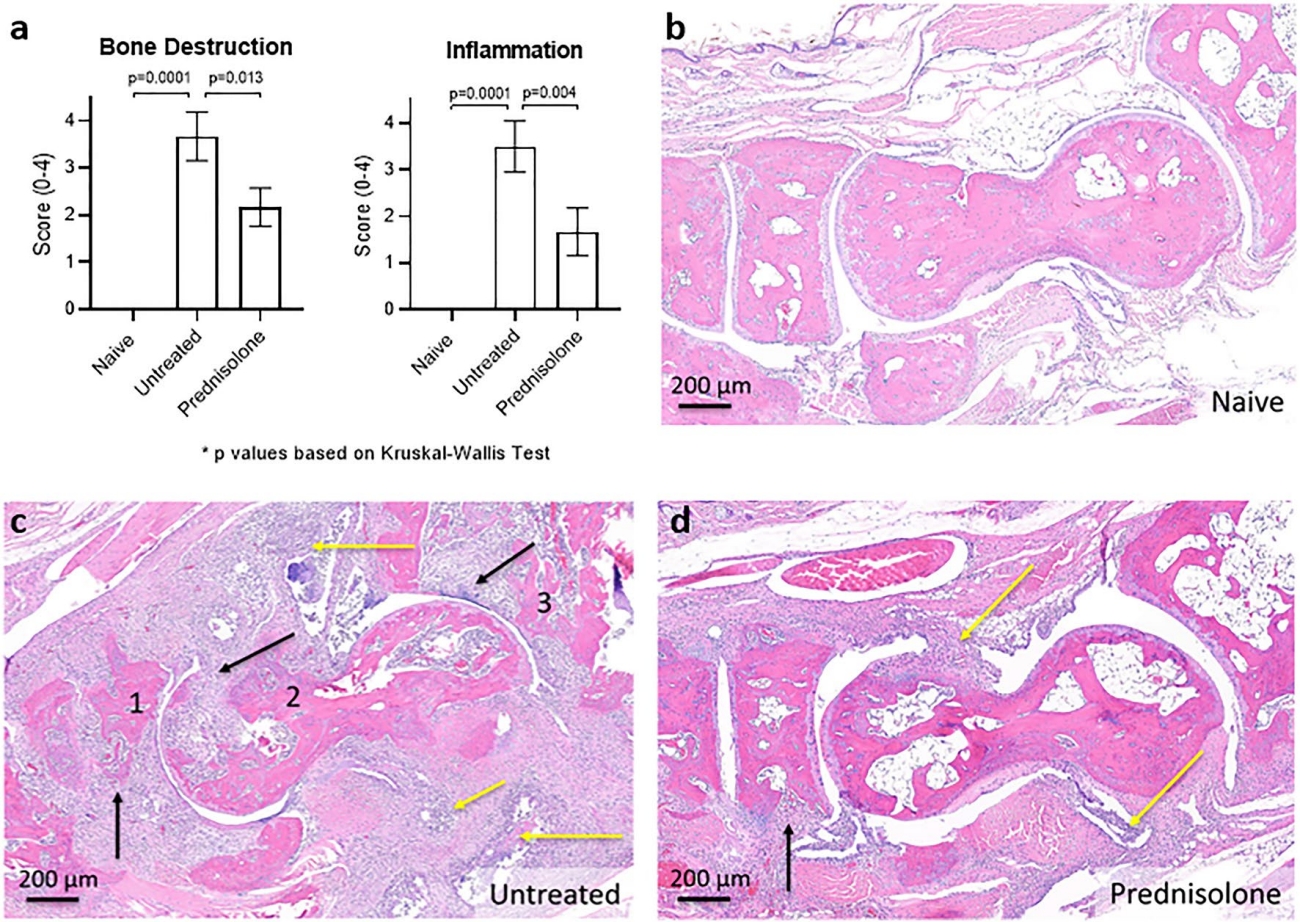
(**a**) Histopatholology scores of tarsal inflammation and bone destruction confirm disease in CIA mice, and show a significant reduction with prednisolone treatment. (**b–d**) Representative hematoxylin and eosin-stained images for each group are shown here with × 50  magnification. Black arrows indicate pannus causing bone erosion in the navicular (1), talus (2) and distal tibia (3). Yellow arrows indicate inflammation. The prednisolone-treated group has less inflammation (yellow arrows), and less bone erosion (black arrows; mainly in the navicular bone, while talus and distal tibia are less affected).

Histopathology evaluation of the enrolled tarsus following necropsy on day 21 confirmed that untreated CIA mice had significantly more bone destruction and inflammation compared to naïve controls (p = 0.0001) (Fig. 6). The bone destruction is caused by synovial proliferation forming pannus which invades the joint space and tarsal bones (Fig. 6c). Relative to the untreated group, the prednisolone treatment group showed a significant reduction in bone destruction and inflammation scores (bone destruction p = 0.013, inflammation p = 0.0004)) (Fig. 6a,d). These improved histopathological scores are in agreement with the improved BV measured by in vivo and ex vivo µCT, as well as reduced swelling measured by calipers and STV with in vivo µCT.

## Discussion

While the advantages offered by longitudinal scanning of bone architecture with in vivo µCT are evident^10,18^, there is a fine balance between X-ray scan doses required for accurate bone morphology measurements and biological response to radiation^19^. Therefore prior to assessing treatment-induced changes in CIA mice, it was important to evaluate potential radiation-induced side effects from image acquisition protocols.

The protocol used here resulted in a radiation dose of 1100 mGy during each imaging session. This dose is much lower than the 5000–7000 mGy of radiation found to be the lethal dose in mice (LD50/30)^7^. However, since the repeated scans over a period of 21 days resulted in a cumulative dose of 4400 mGy over the 4 imaging time points, we evaluated bone architecture, hematological changes, and histopathology to identify the settings for the µCT imaging in DBA mice. As shown in Fig. 2 and Supplementary Fig. 3, these radiation doses were found to not impact cortical bone architecture or peripheral blood cell counts in naïve mice (see ESM). However, the large standard deviations in the hematology measurements, although statistically insignificant, leave open the possibility that there are radiation-induced changes in blood counts, but the bone volume measurements and histological readouts suggest that the impact is minimal.

In this work, we evaluate longitudinal treatment response in the mouse CIA model with an intervention of prednisolone. Prednisolone, a glucocorticoid, has been widely used as a steroid to ameliorate inflammation in murine models of RA as well as in RA patients. Consistent with previously reported data from the CIA model, the peak of inflammation begins on day 7 following enrollment^20^. The results of paw swelling (Fig. 3a) suggest that therapeutic daily doses of prednisolone commencing 7 days after detectable clinical onset can reduce paw inflammation to near baseline in the murine CIA model. These caliper measurements are further supported by BV measurements using in vivo and ex vivo µCT and demonstrate the efficacy of prednisolone administered at peak of inflammation in allowing significant amelioration of bone destruction in the tarsal joints. Further, a strong correlation (R-squared value = 0.98) is observed with BV measurements obtained at the terminal day 21 timepoint with ex vivo vs. in vivo µCT (Fig. 4c). Histopathology assessment on day 21 confirmed the presence of significant tarsal bone destruction in untreated animals and signs of improvement with prednisolone, correlating to the improved BV measurements by in vivo and ex vivo µCT. These correlations provide a rationale for designing preclinical studies in rodent RA models to include in vivo µCT where drug effects can be monitored in a longitudinal manner with reduced animal numbers. Traditional assessment of edema and inflammation in animal models of RA is provided by paw caliper measurement, which offers a rapid readout of disease severity, though lacking precision. In this study, we demonstrate the value of STV quantification to measure soft tissue swelling and establish it as a simple extension of the volumetric analysis used in BV calculations with in vivo µCT, without the need for additional image acquisition. The STV analysis is performed over the same VOI delineated for BV measurement and involves setting a threshold range to identify air as background and bone as extraneous tissue so that only the soft tissue is segmented. The STV changes shown in Fig. 3b for the untreated and prednisolone-treated groups are remarkably consistent with the paw caliper thickness shown in Fig. 3a and exhibit the potential of monitoring both STV and BV in a longitudinal manner in the same animals, with each animal offering its own internal baseline control. A similar approach to quantify STV and BV changes in CAIA mice with or without prednisolone has been reported, however the imaging and related analysis were performed using ex vivo paw samples collected at the end of treatment regimen^13^.

To the best of our knowledge, this is the first study reporting quantification of tissue swelling longitudinally in a rodent model in response to a clinically relevant therapeutic. Soft tissue volume measurements provide a readout of tissue swelling without the subjectivity inherent to paw caliper measurements. The integration of bone volume and soft tissue readouts affords valuable information about inflammation and bone erosion in the rodent tarsus, which can be acquired simultaneously through a single CT image acquisition. The findings reported here provide a comprehensive validation of in vivo µCT imaging as an effective means to monitor the longitudinal effect of treatments in murine models of RA.

## Supplementary Information

Supplementary Figures.
