# Supplementary material for: Quantitative in vivo micro-computed tomography for monitoring disease activity and treatment response in a collagen-induced arthritis mouse model
Source: Sci Rep. 2022 Feb 21;12:2863. doi: 10.1038/s41598-022-06837-w (PMC8861028; doi:10.1038/s41598-022-06837-w)
Supplement: Supplementary file 1 — Supplementary Figures. [file 41598_2022_6837_MOESM1_ESM.docx]

Electronic Supplementary Material

**Quantitative *in vivo* micro-computed tomography for monitoring disease activity and treatment response in a collagen-induced arthritis mouse model**

Audrey E. Lord^1^, Liang Zhang^1^, Jamie E. Erickson^1^, Shaughn Bryant^1^, Christine M. Nelson^1^, Stephanie M. Gaudette^2^, Lucy A. Phillips^1^, Annette J. Schwartz Sterman^1^, Soumya Mitra^*1^

^1^AbbVie Bioresearch Center, Worcester, MA

^2^Worcester Technical High School, Worcester, MA

^*^Corresponding Author

Soumya Mitra, Ph.D.

AbbVie Bioresearch Center

Worcester, MA 016015

(508)-688-3439

soumya.mitra@abbvie.com

**Suppl. Fig. 1**

**
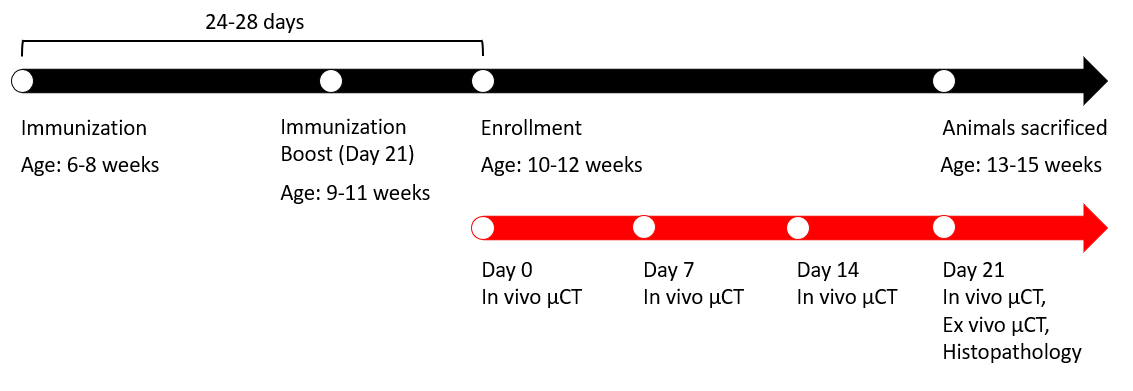
**

A timeline of events pertaining to the study are included here. These time points are relevant for all group except for the naïve group, which had in vivo µCT imaging on days 0, 7, 14, and 28. A subset of the naïve group was imaged using ex vivo µCT and processed for histopathology on Day 0, at 10-12 weeks of age.

**Suppl. Fig. 2**


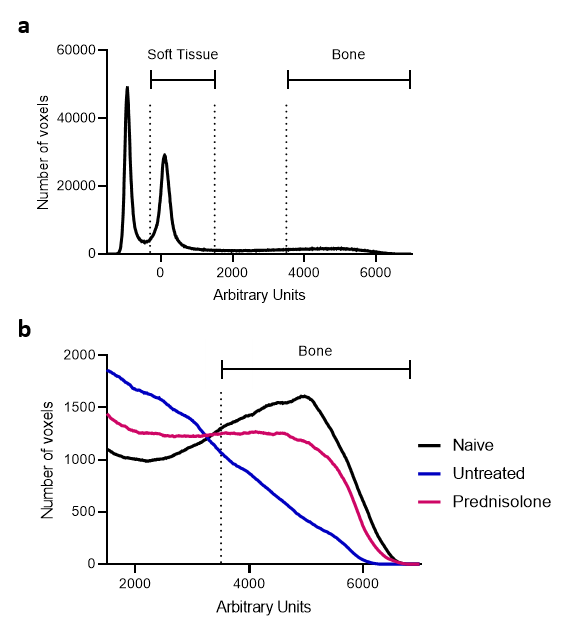


(**a**) A frequency histogram shows the densities of voxels in Arbitrary Units (AU) in an in vivo µCT image. The image is of a representative naïve animal and consists of the same VOI used for other analyses. Vertical dotted lines indicate the density values used for thresholding. Arbitrary Units have been assigned, as opposed to Hounsfield Units (HU), due to the fact that µCT can be linearly calibrated between -1000 HU and 0 HU. However, at far denser ranges such as that of bone, the correction factors and absorption coefficients may be gradually off, leading to an inaccurate representation of HU values in the range of bone. Further, these differences become highly dependent on the scanner. While HU may still be appropriate for soft tissue thresholding, they cannot reliably be assigned here for bone. (**b**) This frequency histogram shows a narrower range of densities to illustrate the changes in bone density associated with disease and treatment on Day 21 of disease. All voxels with a density greater than 3500 AU were attributed to bone. The number of voxels in this density range decreased with disease and prednisolone treatment increased the number of voxels as compared to the untreated group. The formation of bone which is less than 3500 AU can likely be attributed to the formation of periosteal bone which is associated with RA and CIA^2^.

**Suppl. Fig. 3**


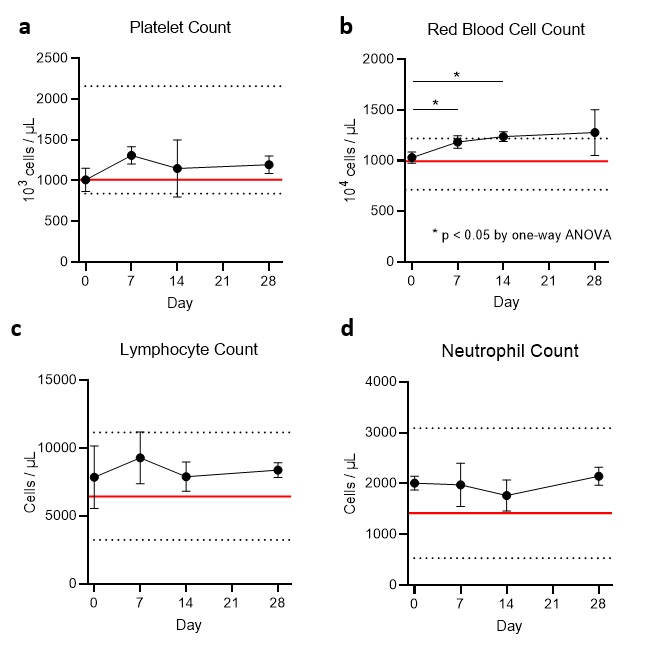


Blood counts are shown for five mice imaged on days 0, 7, 21, and 28. Horizontal black dotted lines represent a standard reference range of values for C57BL/6 mice from Charles River Laboratories (Wilmington, MA) and horizontal red lines indicate the mean values from C57BL/6 and DBA/1J mice as provided by Jackson Laboratory (Bar Harbor, ME). Standard mean values for DBA/1J mice were provided by the vendor, Jackson Laboratories. No statistical differences were detected by one-way ANOVA, with the exception of red blood cell count. (**a**) platelet count (**b**) red blood cell count, (**c**) lymphocyte count, (**d**) neutrophil count.
